# Machine learning selected smoking-associated DNA methylation signatures that predict HIV prognosis and mortality

**DOI:** 10.1186/s13148-018-0591-z

**Published:** 2018-12-13

**Authors:** Xinyu Zhang, Ying Hu, Bradley E. Aouizerat, Gang Peng, Vincent C. Marconi, Michael J. Corley, Todd Hulgan, Kendall J. Bryant, Hongyu Zhao, John H. Krystal, Amy C. Justice, Ke Xu

**Affiliations:** 1 Department of Psychiatry, Yale School of Medicine, 300 George Street, 950 Campbell Ave, West Haven, New Haven, CT 06511 USA; 2 VA Connecticut Healthcare System, 950 Campbell Ave, West Haven, CT 06516 USA; 3 Center for Biomedical Bioinformatics, National Cancer Institute, Rockville, MD 20852 USA; 4 Bluestone Center for Clinical Research, New York University, New York, NY 10010 USA; 5 Department of Biostatistics, Yale School of Public Health, New Haven, CT 065116 USA; 6 Division of Infectious Diseases, Emory University School of Medicine, Atlanta, GA 30303 USA; 7 Department of Native Hawaiian Health, John A. Burns School of Medicine, University of Hawaii, Suite 1016B, Honolulu, 96813 USA; 8 School of Medicine, Vanderbilt University, Nashville, TN 37232 USA; 9 National Institute on Alcohol Abuse and Alcoholism, Bethesda, MD 20852 USA; 10 Yale University School of Medicine, New Haven, CT 06516 USA

**Keywords:** DNA methylation, Ensemble machine learning, HIV frailty, Mortality, Tobacco smoking

## Abstract

**Background:**

The effects of tobacco smoking on epigenome-wide methylation signatures in white blood cells (WBCs) collected from persons living with HIV may have important implications for their immune-related outcomes, including frailty and mortality. The application of a machine learning approach to the analysis of CpG methylation in the epigenome enables the selection of phenotypically relevant features from high-dimensional data. Using this approach, we now report that a set of smoking-associated DNA-methylated CpGs predicts HIV prognosis and mortality in an HIV-positive veteran population.

**Results:**

We first identified 137 epigenome-wide significant CpGs for smoking in WBCs from 1137 HIV-positive individuals (*p* < 1.70E−07). To examine whether smoking-associated CpGs were predictive of HIV frailty and mortality, we applied ensemble-based machine learning to build a model in a training sample employing 408,583 CpGs. A set of 698 CpGs was selected and predictive of high HIV frailty in a testing sample [(area under curve (AUC) = 0.73, 95%CI 0.63~0.83)] and was replicated in an independent sample [(AUC = 0.78, 95%CI 0.73~0.83)]. We further found an association of a DNA methylation index constructed from the 698 CpGs that were associated with a 5-year survival rate [HR = 1.46; 95%CI 1.06~2.02, *p* = 0.02]. Interestingly, the 698 CpGs located on 445 genes were enriched on the integrin signaling pathway (*p* = 9.55E−05, false discovery rate = 0.036), which is responsible for the regulation of the cell cycle, differentiation, and adhesion.

**Conclusion:**

We demonstrated that smoking-associated DNA methylation features in white blood cells predict HIV infection-related clinical outcomes in a population living with HIV.

**Electronic supplementary material:**

The online version of this article (10.1186/s13148-018-0591-z) contains supplementary material, which is available to authorized users.

## Background

Smoking is a common and underappreciated contributor to poor outcomes in HIV-infected individuals. The prevalence of smoking among HIV-infected people exceeds 60% [1], and it is an independent risk factor for mortality in treated HIV-infected individuals [2]. Smoking increases the mortality risk among HIV-infected individuals with an odds ratio between 2 and 3 [2, 3]. However, we have little insight into the mechanisms through which smoking contributes to poorer HIV outcomes.

Smoking-associated effects on DNA methylation in white blood cells (WBCs) have been demonstrated through epigenome-wide association studies (EWAS). DNA methylation is an epigenetic mechanism regulating gene expression independent of variation in the DNA sequence. To date, hundreds of CpG sites (i.e., cytosine-guanine dinucleotides), where cytosines can be methylated to form 5-methylcytosine, in WBCs have been associated with smoking status [4], quantity [5], smoking cessation [6], and smoking-related traits or diseases (e.g., oxidative stress level [7], lung cancer [8], chronic inflammatory disease [9]) in the HIV-uninfected population. Indices of DNA methylation constructed from smoking-associated CpG sites have predicted smoking-related lung cancer incidence [10] and oral cancer incidence [11]. A recent study using a smoking DNA methylation index derived from six CpG sites was associated with frailty in aging populations [12]. And finally, smoking-associated CpGs in the blood were reported to predict all-cause mortality [13, 14] and cardiovascular-related mortality [15]. However, smoking-related DNA methylation associations have not been described in HIV-infected populations to date.

The host epigenome is also impacted by HIV infection. We and others recently showed that DNA methylation is associated with HIV infection and HIV-related aging [16–19]. We reported that CpG sites in the promoter of *NLRC5*, a transcriptional activator of major histocompatibility complex class I, were less methylated in samples from HIV-infected persons as compared to samples from HIV-uninfected persons [19]. Epigenetic marks were also associated with cognitive impairment in the HIV-infected population, and the epigenetic clock relates to biological aging in HIV-infected individuals [20]. Taken together, it is reasonable to hypothesize that both smoking and HIV infection have effects on the epigenome that contribute to poor HIV outcomes and an increased risk of mortality.

To select high-dimensional epigenetic data for predicting clinical outcomes is challenging. For this purpose, machine learning has emerged as a powerful tool that enables the discovery of unknown features in the epigenome to predict phenotypes of interest [21]. Machine learning has been successfully applied to select DNA methylation features to identify biomarkers for complex diseases and to predict treatment outcomes [16, 21, 22]. Recently, a kernel machine learning method improved the prediction of cancer prognosis by integrating molecular profiles and clinical predictors [23]. A panel of DNA methylation markers was able to diagnose common cancers with 95% accuracy and identified 29 out of 30 colorectal cancer metastases [24]. In another study, DNA methylation-based learning selected immune response features improved the prediction of better treatment outcomes of chemotherapy and survival for breast cancer patients [25]. Such an approach can be useful to identify biological signatures of HIV-related outcomes influenced by smoking.

In this study, using an ensemble-based machine learning approach, our goal was to select smoking-associated DNA methylation CpGs in the HIV-infected host epigenome and link the selected CpGs to the HIV disease outcomes. The motivation to use ensemble-based learning is that an ensemble approach has advantages to reduce the bias from individual machine learning methods and to improve the stability of prediction performance in an imbalanced sample [26, 27]. We were also interested in understanding the biological significance of the selected features. This study demonstrates that the application of advanced machine learning on methylation features provides evidence of a link between the mechanisms of smoking and smoking-associated adverse HIV outcomes.

## Results

**Fig. 1:**
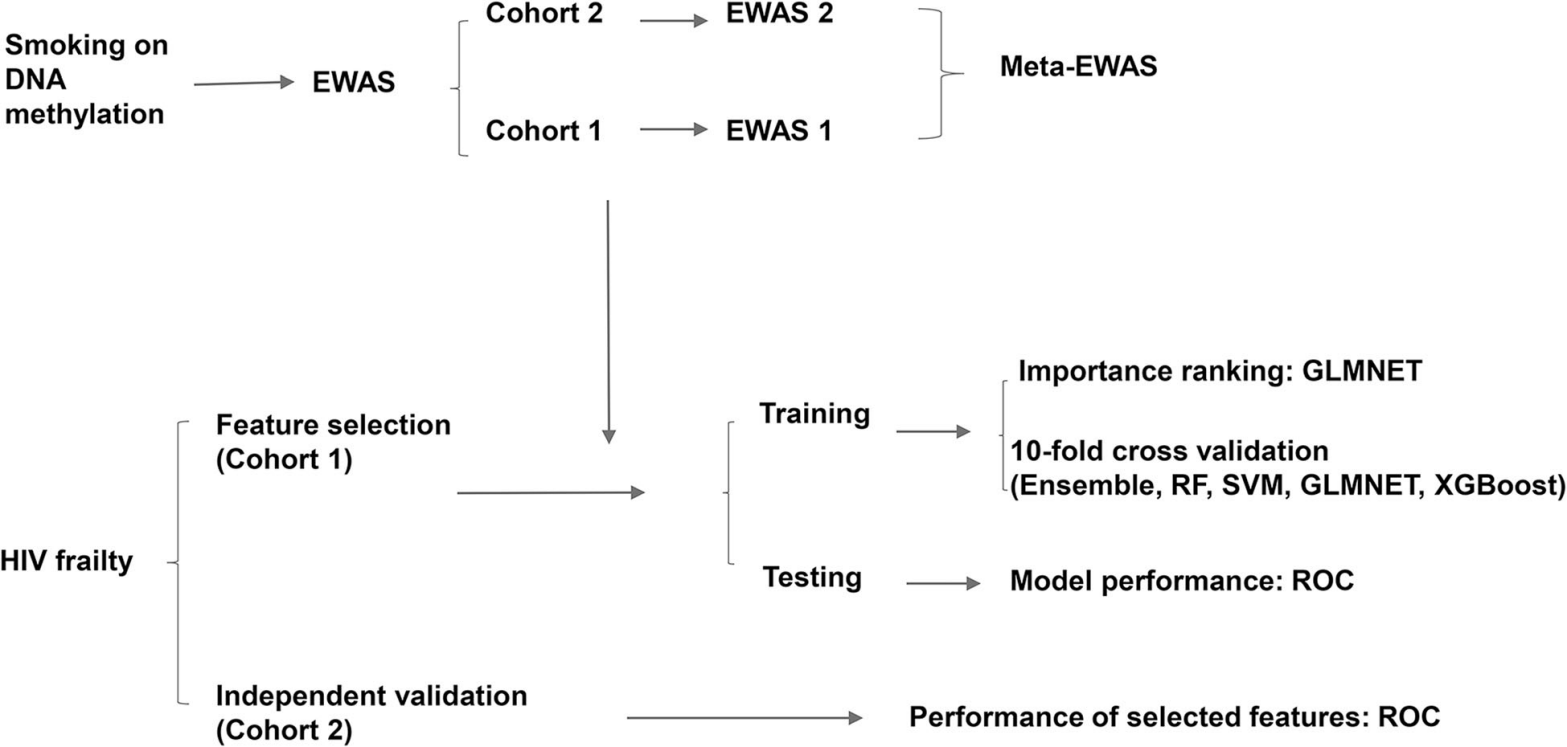
Study design for the epigenome-wide association study for smoking and machine learning prediction

The study design and the framework are presented in Fig. 1. Briefly, all DNA samples were extracted from WBCs collected from people who live with HIV from the Veteran Aging Cohort Study (VACS) (*N* = 1137). All samples were randomly divided into a discovery (cohort 1) sample and a replication (cohort 2) sample. Demographic and clinical variables are presented in Table 1. We first conducted a meta-analysis of the EWAS for smoking in two separate HIV-infected samples. We then selected smoking-associated CpGs that predicted HIV outcomes by using an ensemble-based learning approach.

**Table 1:** Demographics and clinical variables in the study population

	Cohort 1 (Illumina 450K)			Cohort 2 (Illumina Epic)		
	Smokers (*N* = 361)	Non-smokers (*N* = 247)	*p*	Smokers (*N* = 309)	Non-smokers (*N* = 220)	*p*
HIV-positive (%)	100	100	N/A	100	100	N/A
Age	49.2 ± 6.74	49.7 ± 8.70	0.44	48.0 ± 6.55	48.3 ± 9.20	0.62
Sex (male, %)	100	100	N/A	100	100	N/A
Race (AA, %)	87.5	83.4	0.01	79	82.7	0.32
AUDIT-C	4.00 ± 3.32	3.72 ± − 3.48	0.31	3.67 ± 3.05	2.99 ± 2.89	0.01
ART (%)	76.5	76.1	0.87	69.6	83.2	0.0004
WBC	5.35 ± 2.09	5.34 ± 1.81	0.95	5.27 ± 1.96	5.27 ± 1.55	0.99
CD4	408 ± 290	457 ± 272	0.04	415 ± 200	493 ± 280	0.002
VL (log10)	2.75 ± 1.24	2.52 ± 1.15	0.03	2.82 ± 1.26	2.55 ± 1.20	0.02
CD8*	0.18 ± 0.08	0.17 ± 0.08	0.40	0.16 ± 0.08	0.17 ± 0.08	0.38
CD4*	0.05 ± 0.05	0.05 ± 0.05	0.33	0.07 ± 0.05	0.08 ± 0.06	0.19
Nature killer cells*	0.06 ± 0.05	0.09 ± 0.07	< 0.0005	0.08 ± 0.05	0.09 ± 0.06	< 0.005
B cell*	0.09 ± 0.05	0.08 ± 0.05	0.47	0.11 ± 0.05	0.11 ± 0.04	0.44
Monocytes*	0.12 ± 0.04	0.12 ± 0.04	0.83	0.11 ± 0.04	0.10 ± 0.04	0.002
Granulocytes*	0.53 ± 0.13	0.51 ± 0.12	0.07	0.51 ± 0.11	0.49 ± 0.11	0.02

*AA* African-American, *AUDIT* Alcohol Use Disorder Identification Test, *ART* antiretroviral therapy, *VL* viral load

*Methylation-based cell type deconvolution by Housman et al. algorithm

### DNA methylation in WBCs associated with tobacco smoking

#### Discovery

**Fig. 2:**
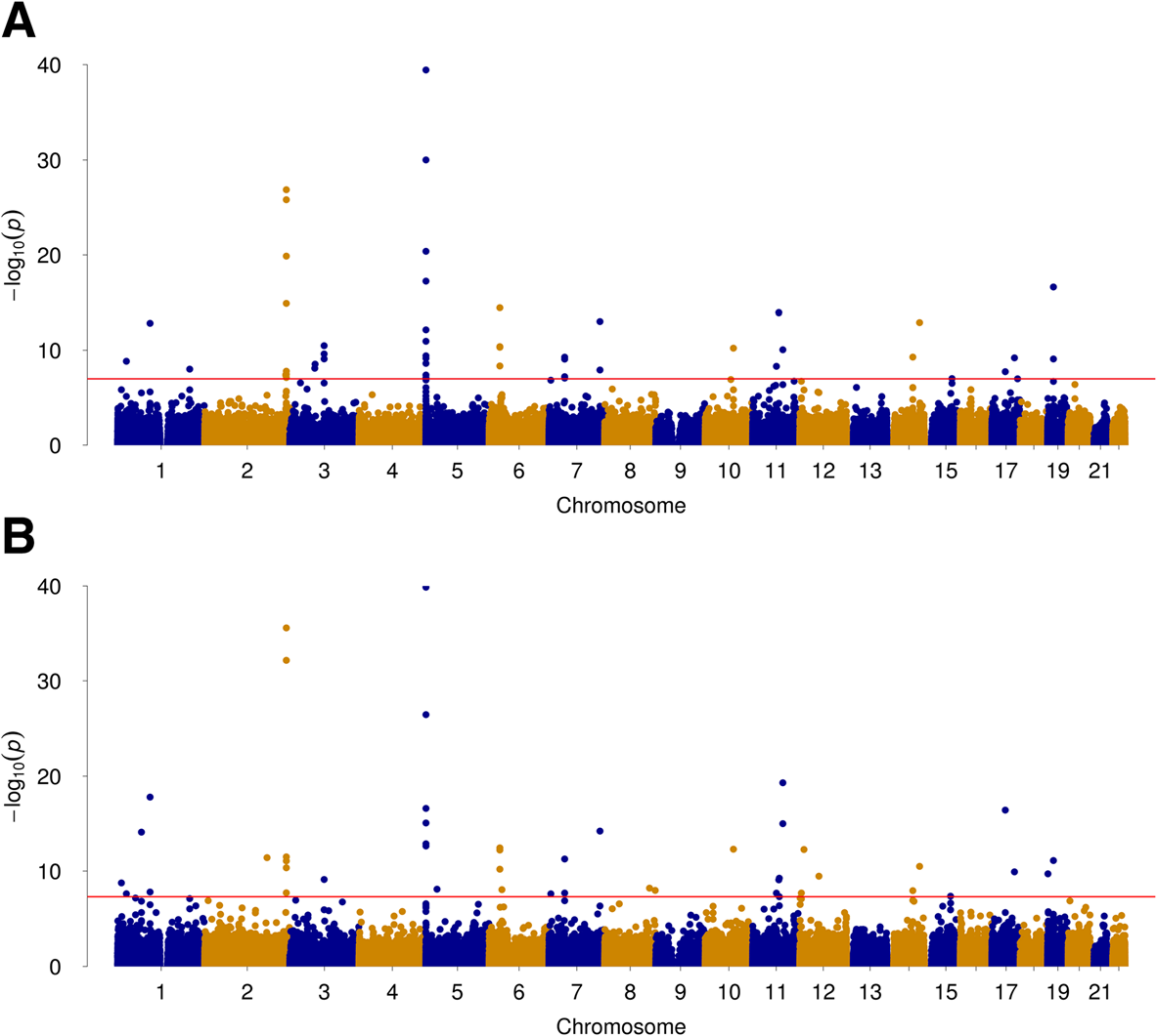
Epigenome-wide association analysis in blood identified multiple CpG sites for tobacco smoking. **a** Discovery sample. **b** Replication sample

We profiled CpGs using the Illumina Infinium HumanMethylation 450 Beadchip (450K) (San Diego, CA, USA) in HIV-infected samples (cohort 1, *N* = 608; current smokers = 361; non-smokers = 247) from the VACS. After adjustment for potential confounders (i.e., age, immune cell types, adherence of antiretroviral therapy, the top principal components to limit global confounding effects), we identified 41 CpGs differentially methylated (i.e., 33 hypomethylated CpGs, 8 hypermethylated CpGs) between smokers and non-smokers (Fig. 2a, *p*_nominal_ < 1.0E−7) (Additional file 1: Table S1). Of note, 40 out of 41 CpG sites were previously reported to be associated with smoking [4, 9, 10, 28–35]. The most significant sites included the established smoking biomarkers on *AHRR* (cg05575921, cg23576855, cg26703534, cg21161138) and on *F2RL3* (cg03636183). One CpG site, cg15212292 located in the body of *PRKCA*, was previously reported significant association for smoking in a large meta-analysis from combined European-American (EA) and African-American (AA) populations but showed no association with smoking in AA [35]. We found this CpG site highly significant in our sample of predominantly AA (*t* = − 8.911; *p* = 5.074E−19). Consistent with previous reports, the majority of smoking-associated CpGs were hypomethylated in smokers as compared to non-smokers.

#### Replication

We conducted a second EWAS for smoking in a sample that was independent of the discovery sample (VACS cohort 2, *N* = 529; current smokers = 309; non-smokers = 220). DNA methylation in the replication sample was profiled using the Illumina Methylation EPIC platform (San Diego, CA, USA) that included 870 K CpGs, with 408,583 CpGs shared between the Illumina 450K and EPIC arrays. To ensure consistency in comparisons across the samples, only CpGs shared across both arrays were assessed. The methylation state probes common to both platforms were highly correlated (*r* ~ 0.91 to 0.99).

Applying the same analytical protocol, we adjusted for the same confounders in the discovery and replication samples. A total of 49 CpG sites reached epigenome-wide significance in the replication sample including the 41 CpGs identified in the discovery EWAS and 8 significant CpGs that were only seen in the replication sample (Fig. 2b) (Additional file 1: Table S2). The 8 additional CpGs were all hypomethylated in smokers compared to non-smokers. The high concordance in findings between the two samples suggests that smoking-associated CpG sites are highly reproducible.

#### Meta-analysis

Combining the discovery and replication samples, a meta-EWAS revealed a total of 137 CpGs that were significantly associated with smoking (*p* < 1.0E−7) (Table 2, Additional file 2: Figure S1). A test for heterogeneity across the two samples for these 137 CpG sites was not significant after Bonferroni correction(*p*_adjusted_ > 0.05) for any of the sites, suggesting that their association with smoking is not due to the confound of sample heterogeneity. Of the 137 CpG sites, 122 sites were hypomethylated, and only 15 CpG sites were hypermethylated in smokers compared to non-smokers. As expected, the most significant CpG site was cg05575921 at *AHRR*. An additional 15 CpG sites on *AHRR* were also significantly associated with smoking status. Consistent with the findings from more than 30 previous studies in HIV-uninfected samples, these results demonstrate that alteration of DNA methylation is associated with smoking exposure regardless of HIV status.

**Table 2:** Epigenome-wide significant CpG sites for tobacco smoking in a veteran HIV-positive population: a meta-analysis

Probe	Chr	Position	Nearest gene	*p*_dis_	Effect (SE)_dis_	*p*_rep_	Effect (SE)_rep_	*Z* score	*p*_meta_	Direction	Hat *p*
cg05575921	5	373,378	*AHRR*	3.64E−40	− 0.134 (0.009)	1.36E−40	− 0.164 (0.011)	− 18.800	7.56E−79	–	0.480
cg21566642	2	233,284,661		1.35E−27	− 0.078 (0.007)	2.64E−36	− 0.096 (0.007)	− 16.543	1.81E−61	–	0.076
cg01940273	2	233,284,934		1.53E−26	− 0.056 (0.005)	6.92E−33	− 0.068 (0.005)	− 15.944	3.12E−57	–	0.144
cg23576855	5	373,299	*AHRR*	9.77E−31	− 0.117 (0.009)	3.67E−27	− 0.127 (0.011)	− 15.791	3.58E−56	–	0.975
cg26703534	5	377,358	*AHRR*	4.18E−21	− 0.034 (0.003)	2.50E−17	− 0.038 (0.004)	− 12.670	8.65E−37	–	0.811
cg21161138	5	399,360	*AHRR*	5.82E−18	− 0.034 (0.004)	8.62E−16	− 0.039 (0.005)	− 11.803	3.78E−32	–	0.994
cg09935388	1	92,947,588	*GFI1*	1.47E−13	− 0.059 (0.008)	1.61E−18	− 0.090 (0.010)	− 11.394	4.48E−30	–	0.167
cg03636183	19	17,000,585	*F2RL3*	2.42E−17	− 0.067 (0.008)	7.86E−12	− 0.056 (0.008)	− 10.861	1.76E−27	–	0.438
cg21322436	7	145,812,842	*CNTNAP2*	9.60E−14	− 0.024 (0.003)	6.16E−15	− 0.028 (0.003)	− 10.766	4.98E−27	–	0.532
cg03329539	2	233,283,329		1.29E−15	− 0.029 (0.003)	3.22E−12	− 0.030 (0.004)	− 10.600	3.00E−26	–	0.720
cg25648203	5	395,444	*AHRR*	7.14E−13	− 0.025 (0.003)	2.29E−13	− 0.033 (0.004)	− 10.248	1.20E−24	–	0.642
cg11660018	11	86,510,915	*PRSS23*	8.89E−11	− 0.024 (0.004)	1.02E−15	− 0.036 (0.004)	− 10.215	1.69E−24	–	0.148
cg23771366	11	86,510,998	*PRSS23*	4.21E−07	− 0.020 (0.004)	4.97E−20	− 0.042 (0.004)	− 9.951	2.50E−23	–	0.001
cg05284742	14	93,552,128	*ITPK1*	1.24E−13	− 0.018 (0.002)	3.22E−11	− 0.020 (0.003)	− 9.947	2.60E−23	–	0.839
cg19572487	17	38,476,024	*RARA*	1.82E−08	− 0.024 (0.004)	3.79E−17	− 0.040 (0.005)	− 9.858	6.30E−23	–	0.020
cg01901332	11	75,031,054	*ARRB1*	1.16E−14	− 0.031 (0.004)	8.50E−10	− 0.030 (0.005)	− 9.830	8.32E−23	–	0.436
cg24859433	6	30,720,203		4.20E−11	− 0.024 (0.004)	3.64E−13	− 0.032 (0.004)	− 9.782	1.35E−22	–	0.415
cg15342087	6	30,720,209		4.85E−11	− 0.023 (0.003)	6.14E−13	− 0.027 (0.004)	− 9.718	2.54E−22	–	0.437
cg03450842	10	80,834,947	*ZMIZ1*	6.08E−11	− 0.018 (0.003)	5.02E−13	− 0.026 (0.003)	− 9.712	2.69E−22	–	0.412
cg04551776	5	393,366	*AHRR*	7.00E−10	− 0.017 (0.003)	1.34E−13	− 0.029 (0.004)	− 9.558	1.20E−21	–	0.227
cg12803068	7	45,002,919	*MYO1G*	8.63E−10	0.047 (0.008)	5.40E−12	0.064 (0.009)	9.188	4.02E−20	++	0.391
cg15212295	17	64,710,687	*PRKCA*	6.36E−10	− 0.014 (0.002)	1.22E−10	− 0.021 (0.003)	− 8.911	5.07E−19	–	0.624
cg14753356	6	30,720,108		4.55E−09	− 0.018 (0.003)	6.42E−11	− 0.028 (0.004)	− 8.744	2.25E−18	–	0.436
cg25189904	1	68,299,493	*GNG12*	3.14E−06	− 0.035 (0.008)	7.95E−15	− 0.066 (0.008)	− 8.708	3.11E−18	–	0.012
cg02657160	3	98,311,063	*CPOX*	8.20E−10	− 0.014 (0.002)	7.79E−10	− 0.015 (0.002)	− 8.685	3.79E−18	–	0.758
cg13193840	2	233,285,289		1.62E−08	− 0.013 (0.002)	4.55E−11	− 0.021 (0.003)	− 8.622	6.58E−18	–	0.336
cg07986378	12	11,898,284	*ETV6*	1.54E−06	− 0.024 (0.005)	5.41E−13	− 0.040 (0.005)	− 8.435	3.30E−17	–	0.046
cg22851561	14	74,214,183	*C14orf43*	5.28E−10	− 0.019 (0.003)	1.17E−08	− 0.026 (0.004)	− 8.432	3.39E−17	–	0.948
cg27537125	1	25,349,681		1.45E−09	− 0.009 (0.002)	2.48E−08	− 0.010 (0.002)	− 8.226	1.93E−16	–	0.960
cg06644428	2	233,284,112		2.07E−06	− 0.016 (0.003)	8.58E−12	− 0.029 (0.004)	− 8.129	4.34E−16	–	0.079
cg14624207	11	68,142,198	*LRP5*	4.95E−09	− 0.015 (0.003)	2.13E−08	− 0.019 (0.003)	− 8.098	5.61E−16	–	0.915
cg26271591	2	178,125,956	*NFE2L2*	5.49E−06	− 0.025 (0.005)	3.90E−12	− 0.052 (0.007)	− 8.058	7.76E−16	–	0.048
cg11902777	5	368,843	*AHRR*	3.87E−10	− 0.012 (0.002)	5.52E−07	− 0.008 (0.002)	− 7.993	1.32E−15	–	0.543
cg19859270	3	98,251,294	*GPR15*	2.46E−10	− 0.013 (0.002)	1.21E−06	− 0.007 (0.001)	− 7.939	2.03E−15	–	0.443
cg08709672	1	206,224,334	*AVPR1B*	9.81E−09	− 0.021 (0.004)	8.12E−08	− 0.019 (0.004)	− 7.852	4.09E−15	–	0.991
cg23916896	5	368,804	*AHRR*	1.14E−11	− 0.040 (0.006)	2.89E−05	− 0.019 (0.004)	− 7.816	5.45E−15	–	0.116
cg19089201	7	45,002,287	*MYO1G*	7.39E−08	0.027 (0.005)	2.09E−08	0.042 (0.007)	7.758	8.63E−15	++	0.669
cg02583484	12	54,677,008	*HNRNPA1*	2.94E−06	− 0.013 (0.003)	3.50E−10	− 0.023 (0.004)	− 7.699	1.37E−14	–	0.162
cg09022230	7	5,457,225	*TNRC18*	1.45E−07	− 0.019 (0.004)	2.52E−08	− 0.027 (0.005)	− 7.646	2.07E−14	–	0.626
cg07178945	12	4,488,800	*FGF23*	1.87E−07	0.020 (0.004)	2.08E−08	0.028 (0.005)	7.635	2.27E−14	++	0.587
cg04885881	1	11,123,118		1.46E−06	− 0.025 (0.005)	1.79E−09	− 0.034 (0.006)	− 7.626	2.43E−14	–	0.265
cg25949550	7	145,814,306	*CNTNAP2*	1.21E−08	− 0.011 (0.002)	4.80E−07	− 0.013 (0.002)	− 7.601	2.94E−14	–	0.837
cg00073090	19	1,265,879		1.20E−05	− 0.011 (0.002)	1.97E−10	− 0.017 (0.003)	− 7.542	4.63E−14	–	0.095
cg27241845	2	233,250,370		2.53E−06	− 0.019 (0.004)	1.98E−08	− 0.034 (0.006)	− 7.270	3.59E−13	–	0.371
cg18900812	6	36,646,127	*CDKN1A*	4.74E−06	− 0.017 (0.004)	9.22E−09	− 0.021 (0.004)	− 7.265	3.74E−13	–	0.280
cg15159987	19	17,003,890	*CPAMD8*	8.33E−10	− 0.019 (0.003)	1.11E−04	− 0.014 (0.004)	− 7.125	1.04E−12	–	0.174
cg19254163	11	60,623,782	*GPR44*	6.38E−07	− 0.012 (0.002)	1.02E−06	− 0.019 (0.004)	− 6.975	3.06E−12	–	0.859
cg08149865	8	21,914,600	*EPB49*	1.23E−06	− 0.014 (0.003)	9.45E−07	− 0.016 (0.003)	− 6.891	5.53E−12	–	0.782
cg20295214	1	206,226,794	*AVPR1B*	1.47E−06	− 0.014 (0.003)	9.65E−07	− 0.011 (0.002)	− 6.863	6.76E−12	–	0.766
cg23219570	12	4,488,893	*FGF23*	1.30E−05	0.012 (0.003)	8.52E−08	0.019 (0.004)	6.842	7.83E−12	++	0.346
cg26529655	5	424,371	*AHRR*	2.39E−06	− 0.011 (0.002)	7.37E−07	− 0.012 (0.002)	− 6.827	8.68E−12	–	0.687
cg11554391	5	321,320	*AHRR*	6.57E−06	− 0.012 (0.003)	2.79E−07	− 0.015 (0.003)	− 6.800	1.05E−11	–	0.495
cg25305703	8	128,378,218		1.26E−04	− 0.020 (0.005)	6.37E−09	− 0.037 (0.006)	− 6.765	1.34E−11	–	0.103
cg09099830	16	30,485,485	*ITGAL*	1.42E−06	− 0.015 (0.003)	2.15E−06	− 0.017 (0.003)	− 6.759	1.39E−11	–	0.860
cg10750182	10	73,497,514	*CDH23*	1.23E−07	− 0.012 (0.002)	2.98E−05	− 0.011 (0.003)	− 6.715	1.88E−11	–	0.579
cg14580211	5	150,161,299	*C5orf62*	1.05E−05	− 0.020 (0.004)	3.19E−07	− 0.030 (0.006)	− 6.709	1.96E−11	–	0.464
cg04180046	7	45,002,736	*MYO1G*	2.39E−05	0.027 (0.006)	1.35E−07	0.045 (0.008)	6.686	2.30E−11	++	0.330
cg17287155	5	393,347	*AHRR*	1.43E−05	− 0.010 (0.002)	3.74E−07	− 0.011 (0.002)	− 6.639	3.15E−11	–	0.450
cg14316231	8	41,895,100	*MYST3*	2.32E−05	− 0.012 (0.003)	2.87E−07	− 0.026 (0.005)	− 6.595	4.26E−11	–	0.386
cg03604011	5	400,201	*AHRR*	1.38E−07	0.012 (0.002)	7.92E−05	0.005 (0.001)	6.544	5.98E−11	++	0.479
cg09662411	1	92,946,132	*GFI1*	3.82E−05	− 0.025 (0.006)	3.56E−07	− 0.038 (0.007)	− 6.484	8.93E−11	–	0.361
cg24996979	14	74,223,355	*C14orf43*	1.17E−04	− 0.009 (0.002)	1.20E−07	− 0.015 (0.003)	− 6.428	1.29E−10	–	0.214
cg13751113	11	118,085,214	*AMICA1*	8.66E−06	− 0.011 (0.003)	5.27E−06	− 0.015 (0.003)	− 6.359	2.03E−10	–	0.767
cg00310412	15	74,724,918	*SEMA7A*	3.66E−04	− 0.011 (0.003)	4.49E−08	− 0.020 (0.004)	− 6.337	2.34E−10	–	0.117
cg18642234	3	49,394,622	*GPX1*	1.24E−06	− 0.013 (0.003)	4.44E−05	− 0.012 (0.003)	− 6.331	2.43E−10	–	0.748
cg26361535	8	144,576,604	*ZC3H3*	9.58E−04	− 0.014 (0.004)	1.10E−08	− 0.029 (0.005)	− 6.313	2.74E−10	–	0.054
cg00295485	2	106,755,721	*UXS1*	5.86E−05	− 0.017 (0.004)	7.63E−07	− 0.029 (0.006)	− 6.311	2.77E−10	–	0.382
cg03440944	7	45,023,329	*C7orf40*	8.68E−08	− 0.012 (0.002)	4.66E−04	− 0.013 (0.004)	− 6.301	2.96E−10	–	0.275
cg12075928	8	141,801,307	*PTK2*	4.90E−06	− 0.021 (0.005)	1.60E−05	− 0.026 (0.006)	− 6.284	3.30E−10	–	0.969
cg01481251	11	32,912,719		5.67E−05	− 0.016 (0.004)	1.05E−06	− 0.012 (0.002)	− 6.274	3.51E−10	–	0.410
cg03707168	19	49,379,127	*PPP1R15A*	1.16E−05	− 0.024 (0.006)	6.89E−06	− 0.027 (0.006)	− 6.274	3.52E−10	–	0.766
cg11436113	20	19,191,145		1.29E−05	− 0.014 (0.003)	6.14E−06	− 0.018 (0.004)	− 6.274	3.53E−10	–	0.741
cg00706683	2	233,251,030	*ECEL1P2*	7.54E−08	0.020 (0.004)	6.22E−04	0.016 (0.005)	6.267	3.69E−10	++	0.244
cg10062919	17	38,503,802	*RARA*	3.21E−05	− 0.008 (0.002)	2.37E−06	− 0.009 (0.002)	− 6.259	3.86E−10	–	0.539
cg06394460	13	28,130,393	*LNX2*	8.32E−07	− 0.028 (0.006)	1.36E−04	− 0.019 (0.005)	− 6.206	5.44E−10	–	0.568
cg04517079	6	41,546,161	*FOXP4*	1.28E−04	− 0.010 (0.002)	6.03E−07	− 0.014 (0.003)	− 6.205	5.48E−10	–	0.300
cg16547579	20	4,954,333	*SLC23A2*	3.74E−04	− 0.011 (0.003)	1.39E−07	− 0.019 (0.004)	− 6.194	5.87E−10	–	0.154
cg21446172	1	223,745,234	*CAPN8*	1.76E−04	− 0.010 (0.003)	4.68E−07	− 0.020 (0.004)	− 6.180	6.40E−10	–	0.260
cg07251887	17	73,641,809	*RECQL5*	1.03E−07	− 0.016 (0.003)	8.91E−04	− 0.013 (0.004)	− 6.158	7.38E−10	–	0.230
cg15474579	6	36,645,812	*CDKN1A*	1.01E−05	− 0.013 (0.003)	2.32E−05	− 0.018 (0.004)	− 6.115	9.66E−10	–	0.934
cg16398761	14	74,220,238	*C14orf43*	1.58E−05	− 0.010 (0.002)	2.13E−05	− 0.013 (0.003)	− 6.056	1.39E−09	–	0.870
cg13039251	5	32,018,601	*PDZD2*	4.03E−03	0.014 (0.005)	8.21E−09	0.040 (0.007)	6.035	1.59E−09	++	0.024
cg23110422	21	40,182,073	*ETS2*	5.94E−05	− 0.015 (0.004)	5.59E−06	− 0.020 (0.004)	− 6.034	1.60E−09	–	0.560
cg02275418	3	15,372,726	*SH3BP5*	5.16E−05	− 0.009 (0.002)	7.61E−06	− 0.008 (0.002)	− 6.013	1.82E−09	–	0.609
cg03188382	2	233,245,886	*ALPP*	3.42E−08	− 0.021 (0.004)	3.83E−03	− 0.012 (0.004)	− 6.008	1.88E−09	–	0.099
cg14712058	19	16,988,083	*SIN3B*	1.93E−07	− 0.018 (0.003)	1.37E−03	− 0.012 (0.004)	− 5.990	2.09E−09	–	0.226
cg03109660	4	37,684,505	*RELL1*	4.91E−06	− 0.013 (0.003)	1.05E−04	− 0.018 (0.005)	− 5.987	2.14E−09	–	0.780
cg08035323	2	9,843,525		1.19E−03	0.017 (0.005)	1.31E−07	0.036 (0.007)	5.970	2.37E−09	++	0.099
cg13038618	14	77,467,391		1.08E−03	− 0.011 (0.003)	1.54E−07	− 0.020 (0.004)	− 5.970	2.38E−09	–	0.108
cg03234777	11	118,095,544	*AMICA1*	4.97E−05	− 0.009 (0.002)	1.34E−05	− 0.011 (0.002)	− 5.936	2.92E−09	–	0.677
cg13422817	12	4,550,927	*FGF6*	1.02E−05	0.011 (0.003)	7.28E−05	0.013 (0.003)	5.933	2.98E−09	++	0.913
cg15657641	11	47,939,769		1.73E−06	0.008 (0.002)	3.77E−04	0.007 (0.002)	5.923	3.17E−09	++	0.508
cg24851513	3	52,099,522	*C3orf74*	1.44E−04	0.007 (0.002)	4.37E−06	0.007 (0.002)	5.913	3.37E−09	++	0.444
cg17485141	2	42,566,556		8.57E−04	0.005 (0.002)	4.17E−07	0.014 (0.003)	5.890	3.86E−09	++	0.154
cg00385142	3	98,235,918	*CLDND1*	2.87E−07	− 0.009 (0.002)	1.81E−03	− 0.004 (0.001)	− 5.881	4.09E−09	–	0.223
cg26764244	1	68,299,511	*GNG12*	1.68E−03	− 0.014 (0.004)	1.52E−07	− 0.026 (0.005)	− 5.878	4.14E−09	–	0.090
cg01899089	5	369,969	*AHRR*	4.09E−08	− 0.016 (0.003)	6.94E−03	− 0.011 (0.004)	− 5.854	4.80E−09	–	0.077
cg25013095	2	231,809,672		3.75E−08	− 0.008 (0.001)	7.93E−03	− 0.002 (0.001)	− 5.835	5.39E−09	–	0.070
cg25292882	15	39,431,467		1.72E−04	− 0.011 (0.003)	6.24E−06	− 0.009 (0.002)	− 5.829	5.57E−09	–	0.459
cg14179389	1	92,947,961	*GFI1*	6.93E−03	− 0.013 (0.005)	1.63E−08	− 0.033 (0.006)	− 5.827	5.66E−09	–	0.022
cg09837977	7	110,731,201	*LRRN3*	8.75E−06	− 0.008 (0.002)	1.92E−04	− 0.006 (0.002)	− 5.795	6.84E−09	–	0.760
cg16503724	3	17,130,667	*PLCL2*	2.95E−03	0.007 (0.002)	1.19E−07	0.020 (0.004)	5.786	7.22E−09	++	0.065
cg20454518	12	133,135,463	*FBRSL1*	2.88E−04	0.021 (0.006)	6.45E−06	0.028 (0.006)	5.728	1.01E−08	++	0.409
cg16382047	2	231,790,037	*GPR55*	6.89E−06	− 0.018 (0.004)	3.70E−04	− 0.023 (0.006)	− 5.717	1.08E−08	–	0.643
cg06972908	16	30,488,321	*ITGAL*	1.26E−05	− 0.009 (0.002)	2.17E−04	− 0.013 (0.003)	− 5.716	1.09E−08	–	0.784
cg00605777	2	97,533,635	*SEMA4C*	1.29E−05	− 0.009 (0.002)	2.47E−04	− 0.009 (0.003)	− 5.690	1.27E−08	–	0.768
cg00300637	5	319,433	*AHRR*	9.94E−04	0.012 (0.004)	1.84E−06	0.021 (0.004)	5.661	1.50E−08	++	0.214
cg01882991	6	6,677,756		5.00E−05	− 0.010 (0.002)	8.16E−05	− 0.013 (0.003)	− 5.653	1.58E−08	–	0.909
cg05049335	11	66,103,889	*RIN1*	4.95E−07	− 0.008 (0.002)	4.26E−03	− 0.007 (0.002)	− 5.627	1.84E−08	–	0.180
cg10788371	11	76,381,040	*LRRC32*	6.06E−02	− 0.006 (0.003)	5.69E−10	− 0.024 (0.004)	− 5.600	2.14E−08	–	0.001
cg04716530	16	30,485,684	*ITGAL*	3.32E−05	− 0.006 (0.002)	1.78E−04	− 0.009 (0.002)	− 5.592	2.25E−08	–	0.928
cg09006487	3	72,424,982	*RYBP*	2.93E−09	− 0.023 (0.004)	6.83E−02	− 0.009 (0.005)	− 5.584	2.35E−08	–	0.007
cg10841124	5	433,274	*AHRR*	2.42E−05	0.007 (0.002)	2.63E−04	0.013 (0.004)	5.577	2.45E−08	++	0.833
cg01956154	14	94,423,399	*ASB2*	3.59E−05	− 0.009 (0.002)	1.81E−04	− 0.009 (0.002)	− 5.576	2.46E−08	–	0.936
cg26800893	11	67,184,596	*ATPGD1*	5.39E−05	0.008 (0.002)	1.21E−04	0.008 (0.002)	5.575	2.48E−08	++	0.955
cg05460226	17	8,804,279	*PIK3R5*	2.57E−−04	− 0.015 (0.004)	2.13E−05	− 0.029 (0.007)	− 5.572	2.51E−08	–	0.538
cg18146737	1	92,946,700	*GFI1*	1.99E−04	− 0.036 (0.010)	3.50E−05	− 0.037 (0.009)	− 5.543	2.97E−08	–	0.625
cg16201146	20	19,191,526		4.06E−07	− 0.012 (0.002)	7.10E−03	− 0.009 (0.003)	− 5.541	3.01E−08	–	0.137
cg16814719	8	134,114,834	*TG;SLA*	4.48E−06	− 0.005 (0.001)	1.41E−03	− 0.005 (0.002)	− 5.533	3.16E−08	–	0.427
cg00501876	3	39,193,251	*CSRNP1*	5.22E−04	− 0.009 (0.003)	1.15E−05	− 0.014 (0.003)	− 5.529	3.22E−08	–	0.400
cg08763102	4	3,079,751	*HTT*	1.73E−03	− 0.007 (0.002)	2.13E−06	− 0.015 (0.003)	− 5.525	3.30E−08	–	0.184
cg07381806	19	2,094,327	*MOBKL2A*	1.68E−03	− 0.015 (0.005)	2.62E−06	− 0.035 (0.007)	− 5.502	3.75E−08	–	0.196
cg01731783	14	74,211,788	*C14orf43*	8.41E−07	− 0.008 (0.002)	5.40E−03	− 0.006 (0.002)	− 5.500	3.81E−08	–	0.185
cg19717773	7	2,847,554	*GNA12*	1.86E−04	− 0.033 (0.009)	5.57E−05	− 0.043 (0.011)	− 5.482	4.21E−08	–	0.691
cg17791651	1	38,513,489	*POU3F1*	4.05E−05	− 0.011 (0.003)	2.99E−04	− 0.012 (0.003)	− 5.468	4.55E−08	–	0.876
cg23161492	15	90,357,202	*ANPEP*	6.62E−04	− 0.016 (0.005)	1.30E−05	− 0.026 (0.006)	− 5.464	4.66E−08	–	0.387
cg07465627	17	53,167,407	*STXBP4*	3.03E−06	− 0.011 (0.002)	2.99E−03	− 0.013 (0.004)	− 5.439	5.36E−08	–	0.311
cg16702313	14	74,251,926	*C14orf43*	4.78E−04	− 0.007 (0.002)	2.64E−05	− 0.008 (0.002)	− 5.421	5.94E−08	–	0.490
cg07339236	20	50,312,490	*ATP9A*	5.74E−03	− 0.007 (0.003)	6.65E−07	− 0.014 (0.003)	− 5.411	6.27E−08	–	0.080
cg15187398	19	2,093,896	*MOBKL2A*	2.94E−03	− 0.011 (0.004)	2.10E−06	− 0.019 (0.004)	− 5.410	6.29E−08	–	0.150
cg02743070	10	80,834,309	*ZMIZ1*	7.44E−05	− 0.007 (0.002)	2.35E−04	− 0.009 (0.002)	− 5.406	6.45E−08	–	0.990
cg20059928	15	40,361,485		4.01E−05	− 0.025 (0.006)	5.01E−04	− 0.021 (0.006)	− 5.377	7.57E−08	–	0.798
cg14120703	9	139,416,102	*NOTCH1*	4.33E−05	− 0.008 (0.002)	4.80E−04	− 0.008 (0.002)	− 5.372	7.79E−08	–	0.814
cg11816838	3	150,484,093		1.33E−02	− 0.006 (0.002)	1.81E−07	− 0.009 (0.002)	− 5.369	7.90E−08	–	0.033
cg26057754	1	183,774,231	*RGL1*	6.85E−06	− 0.004 (0.001)	2.32E−03	− 0.003 (0.001)	− 5.367	8.01E−08	–	0.400
cg19713851	2	233,246,594	*ALPP*	5.33E−05	− 0.031 (0.008)	4.12E−04	− 0.032 (0.009)	− 5.364	8.13E−08	–	0.863
cg03519879	14	74,227,499	*C14orf43*	8.89E−07	− 0.008 (0.002)	9.74E−03	− 0.006 (0.002)	− 5.357	8.46E−08	–	0.144

### Ensemble-based feature selection of DNA methylation for HIV frailty

The VACS index was used as an indicator of HIV outcome [36]. High HIV frailty and poor prognosis was defined as a VACS index of greater than 50. Ensemble learning was applied to classify the samples with a VACS index score of greater than 50 as having a poor prognosis, and samples with a VACS index of less than 50 as having a good prognosis. All samples were divided into a training set (80% of the samples in cohort 1), a testing set (20% of the samples in cohort 1), and a validating set (cohort 2).

We first filtered CpGs based on *p* values (false discovery rate, FDR < 0.5) from the EWAS analysis. A total of 997 candidate CpGs from the discovery EWAS were used for feature selection. The goal of the feature selection was to eliminate redundant and irrelevant CpGs without losing informative loci that were associated with high frailty and poor prognosis. In our sample, the numbers of high and low VACS index samples were unequal (high VACS index = 237, low VACS index = 900). Individual machine learning approaches favor the classification of samples into the larger class (e.g., low VACS index samples). To reduce this potential bias without decreasing the sample employed in the training set, we applied a greedy ensemble-based feature selection to build a classifier less likely to be biased towards the larger class from the four machine learning methods(i.e., lasso and elastic-net regularized generalized linear model (GLMNET), support vector method (SVM), random forest (RF), and XGBoot).

**Fig. 3:**
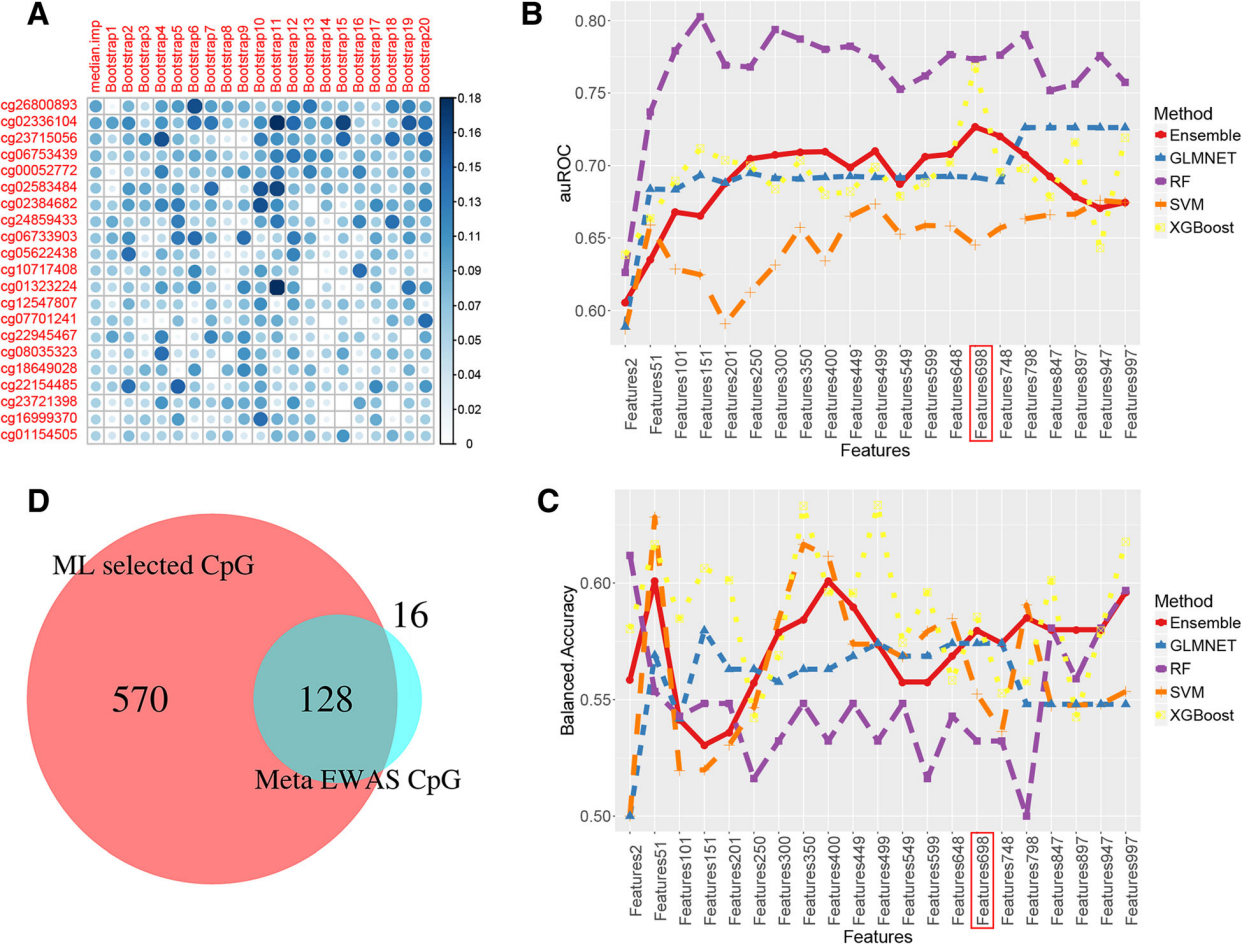
**a** Importance rank in 997 CpG sites using GLMNET method following 100 bootstraps. Only top-ranked 20 CpG sites are displayed. **b** Performance of the selected features predicting high HIV frailty (Veteran Aging Cohort index, VACS index) in a test sample set measured by area under curve (AUC) in receiver operating characteristic analysis. Ensemble-based machine learning from GLMNET, RF, SVM, and XGBoost was applied. **c** Performance of the selected features predicting high HIV frailty (Veteran Aging Cohort index, VACS index) in a test sample set measured by balanced accuracy. Ensemble-based machine learning from four base machine learning methods, GLMNET, RF, SVM, and XGBoost, was applied. **d** Venn plot showing the overlapping CpG sites between the selected 698 features and epigenome-wide significant CpG sites for tobacco smoking

In the *training* sample from cohort 1, we applied a bootstrap aggregating (Bagging) approach, in which GLMNET was used with 100 bootstraps using 70% of the training sample, to weigh the importance of each CpG (Fig. 3a). The CpGs were subsequently clustered into 21 CpG groups from 2 to 997 CpGs based on the importance rank with an incrementation of 50 CpGs. Four machine learning methods, GLMNET, SVM, RF, and XGBoost, were applied to build prediction models using each CpG group separately. Then, a set of classifiers was determined and used to classify new data points by taking a weighted average of the prediction from each of the four machine learning methods. The performance of tenfold cross-validation for each CpG group showed high sensitivity (> 0.9) but relatively low specificity (< 0.5) for each of the 4 machine learning methods. The models from ensemble learning and 4 individual machine learnings were evaluated in the test sample separately.

In the *testing* set, the ensemble method selected a set of 689 CpGs that discriminated poor and good prognosis with the best performance (Fig. 3b). The prediction efficiency was estimated using receiver operator characteristic curves; the 698 CpG set displayed an area under curve (AUC) of 0.73 (95%CI 0.63~0.83) for high HIV frailty. The AUCs from RF and XGBoost at the 698 CpGs were also high (0.76). Although RF and XGBoost had high AUCs across all CpG sets, their balanced accuracy was not as good as ensemble method (Fig. 3c). Therefore, the set of 698 CpGs was selected to test the prediction efficiency. Importantly, the majority of EWAS-significant CpGs (121 out of 137 EWAS-significant CpGs) were included in the 698 CpGs (Fig. 3d), suggesting that ensemble learning enables the selection of biologically informative CpGs to predict HIV frailty.

### Validation of prediction for HIV frailty using the selected 698 CpGs

**Fig. 4:**
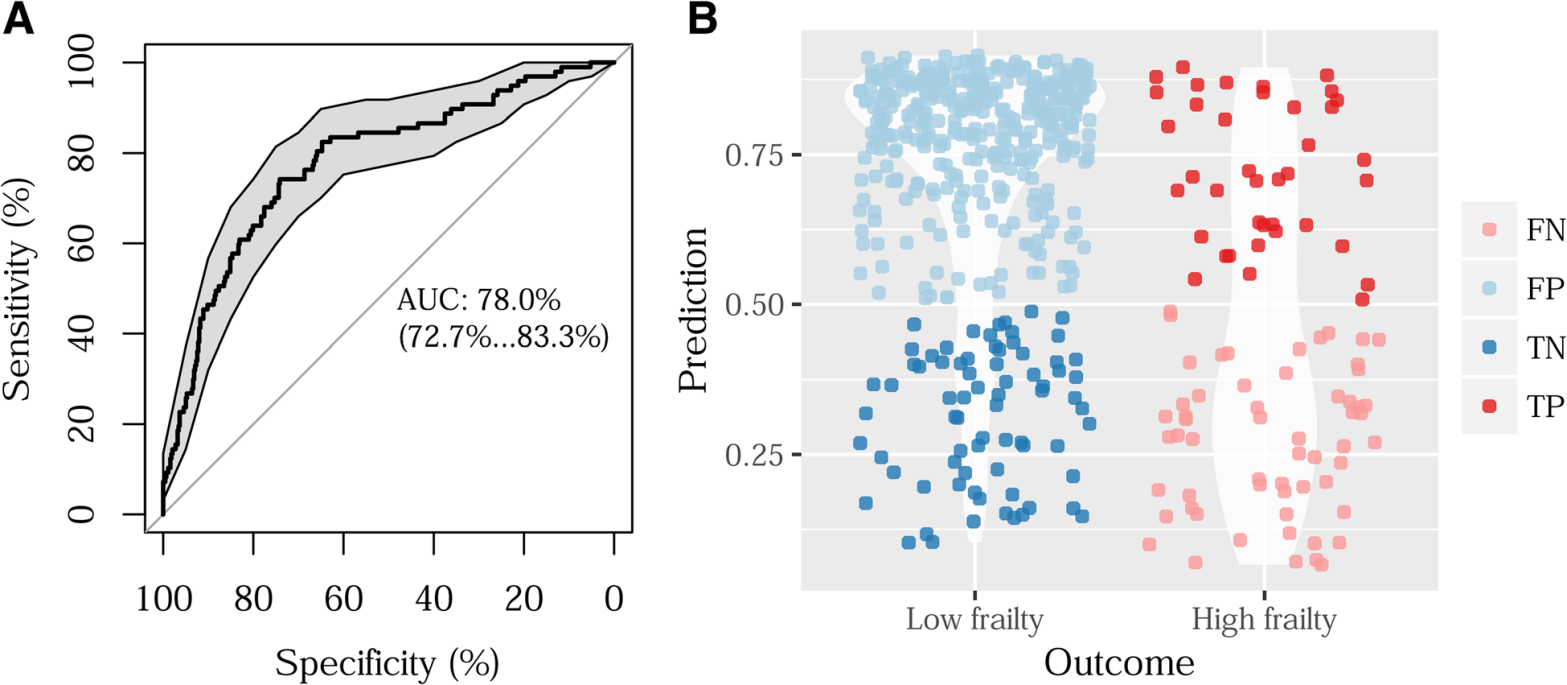
Validation of performance of the selected 698 CpG sites predicting high HIV frailty (Veteran Aging Cohort index, VACS index) in an independent sample set. **a** Area under curve (AUC) from receiver operating characteristic analysis. **b** Sensitivity and specificity of the 698 CpGs predicting the high score of VACS index. FN, false negative; FP, false positive; TN, true negative; TP, true positive

To further validate the prediction results of the 698 CpGs from the discovery sample, we tested the prediction efficiency in the replication sample (cohort 2). Using the same VACS index score cut point, we found that the AUC was 0.78 (95%CI 0.73~0.83) (Fig. 4). The balanced accuracy of prediction was improved to 0.76. The results suggest that the model built in the training set had minimal overfitting features and can be applied to differentiate good and poor HIV prognosis in independent samples.

Of note, to test whether an individual machine learning method alone, a penalized regression model, can select a smaller number of CpG sites than ensemble learning from genome-wide CpG sites to predict HIV frailty, we conducted a feature selection from 408,583 CpG sites using GLMNET to predict the same high and low VACS index. We found that GLMNET selected 1852 CpG sites that predicted the VACS index with AUC of 0.76 (Additional file 3: Figure S2). Although the performance of GLMNET was comparable to the ensemble-based approach, the latter was able to select a smaller number of features and linked smoking-DNA methylation to HIV outcomes.

We tested whether ensemble learning can predict resilient persons that are HIV-positive. Using cutoff of the VACS index < 16 as an excellent prognosis, we found that ensemble learning showed poor performance prediction (AUC < 0.7 and balanced accuracy < 0.5). The poor prediction is likely due to an insufficient number of samples with excellent prognosis (i.e., the sample was underpowered).

We were also interested in understanding whether the prediction of the high and low VACS index using the 698 CpG sites performed better than smoking status alone. We found the AUC of smoking status predicting VACS index was 0.55 (Additional file 4: Figure S3), suggesting that smoking-associated DNA methylation is a better predictor for HIV frailty compared to smoking status alone.

### Prediction of the selected 698 CpGs for all-cause mortality in HIV infection

To support the value of the 698-CpG set in predicting HIV outcomes, the ability of the set to predict mortality in HIV-infected individuals was evaluated. Using the same ensemble model, we first tested the prediction performance of the 698 CpGs with mortality in cohort 2, in which 84 subjects died within 5 years after the blood draw used to profile the DNA methylome. The AUC was 0.66 (95%CI 0.60~0.73) (Additional file 5: Figure S4), which was not as good as the prediction of HIV frailty.

**Fig. 5:**
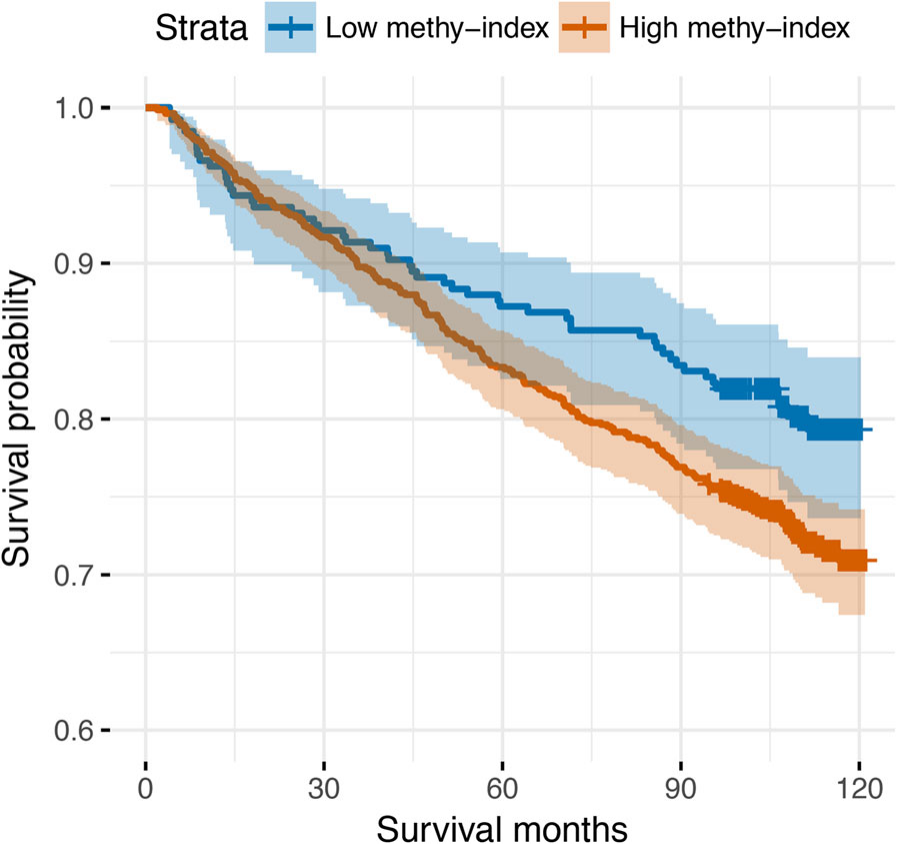
Association of a methylation index constructed from smoking—698 CpG sites with a 5-year survival rate in HIV-infected samples

We then constructed a DNA methylation index score based on the coefficient of each CpG site from the 698 CpGs in cohort 1. After adjusting for confounding factors such as age, CD4 count, viral load, and antiretroviral therapy, we found a significant association between the methylation index and the 5-year survival rate in cohort 2 (HR = 1.46; 95%CI 1.06~2.02, *p* = 0.02) (Fig. 5). As expected, the significant association was driven by hypomethylated CpG sites for smoking (HR = 1.39, *p* = 0.02) but not by hypermethylated CpGs for smoking (HR = 1.21, *p* = 0.21). The results provide further evidence that DNA methylation-based prediction of mortality can be applied in the HIV-infected population.

### Biological significance of the selected 698 CpG sites

The selected 698 CpGs were located among 445 genes (Additional file 1: Table S3). Pathway analysis showed a significant enrichment on the canonical integrin signaling pathway (*p* = 9.55E−05, FDR = 0.036). Fourteen out of 445 genes were in this pathway: *MAP2K4*, *ITGA2B*, *ARHGAP26*, *PIK3R5*, *ITGAL*, *PTK2*, *NCK2*, *CAPN8*, *RHOG*, *GAB1*, *LIMS1*, *ITGA11*, *CTTN*, and *ACTN1*. Integrin signaling determines cellular responses such as migration, survival, differentiation, and motility and provides a context for responding to other inputs. The function of integrin signaling is critical for cell adhesion, tissue maintenance and repair, host defense, and hemostasis. Among non-canonical pathways, cancer, organismal injury, and abnormalities were the most significant (FDR = 1.87E−17). Other top disease-related pathways were in the categories of gastrointestinal disease, liver hyperproliferation, and dermatological diseases. These results suggest that ensemble learning selected biologically relevant features underlying pathological changes in smoking-related diseases.

## Discussion

Applying a DNA methylation-based machine learning approach, we report a set of smoking-associated DNA methylation sites predicting HIV prognosis and mortality in people living with HIV. The prediction of HIV frailty by the selected features showed an ability to accurately differentiate good and poor HIV-related clinical outcomes in an independent sample. The DNA methylation index constructed from the selected CpGs was also associated with mortality in the HIV-infected population. Interestingly, the selected smoking-associated methylation features were enriched in the integrin signaling pathway and related to multiple cancers and organismal injuries, which supports the hypothesis that the contributions of smoking to poor disease outcome are in part due to the changes in DNA methylation in the HIV-infected host epigenome. The study has demonstrated that the application of methylation-based machine learning can be useful for linking molecular information to clinical outcomes.

One of the major challenges to building a successful model using high dimensional data to predict disease outcomes is how to select informative features among redundant or irrelevant data, background noise, and biased features [21]. We applied several approaches to guide the machine learning process. First, epigenome CpGs were filtered based on association analysis of DNA methylation sites with smoking, which considerably reduced the number of features for model building. We rationalized using smoking-associated features because smoking alters DNA methylation, and smokers have higher mortality rates in the population when living with HIV. Second, we applied ensemble learning based on the results of multiple machine learning methods to optimize the selected features and to limit the bias of each method. This data processing method typically improves the accuracy of the model when employing an unbalanced sample. Our results showed that the performance of the ensemble-based model is highly reproducible and better than individual machine learning method such as GLMNET. The advantage of the greedy ensemble machine learning approach can also reduce overfitting and improve model stability [37]. Overfitting is another major challenge in building a predictive model. To address this concern, we split the sample into two cohorts: cohort 1, which was sub-divided into a training set and a testing set, and cohort 2, which was used to replicate the predictive model performance. Thus, the features selected from cohort 1 could be independently tested in cohort 2. Therefore, the steps we took ensured we selected features with high accuracy to predict HIV outcomes.

Our results showed that the selected features were predictive for HIV frailty with moderate to high sensitivity and specificity. Methylation marks for smoking were previously applied to predict frailty in an elderly population. Gao et al. reported that 9 smoking-associated CpG sites were significantly associated with higher frailty. We found that our selected 698 features showed better performance (AUC 0.78 versus AUC 0.55), which may be due to the inclusion of significantly more CpG sites and different populations in our sample compared to the Gao et al. study. The prediction of HIV frailty using the selected 698 features also outperformed the use of tobacco smoking alone.

We found that the prediction of 698 sites for mortality was not as good as the prediction for the VACS index. This result is not unexpected as the model was built for the VACS index, not for mortality. Second, the number of deaths by cohort was unbalanced. In cohort 2, only 87 individuals had died at the time of this analysis, which may have reduced the power for accurate prediction. However, the methylation index with 698 CpGs was significantly predictive for 5-year survival rate. Individuals with a greater methylation index were more likely to have shorter life expectancy than individuals with lower methylation index.

Importantly, the selected DNA methylation features were not only computationally effective for classifying good and poor outcomes and for predicting mortality but were also biologically relevant to HIV frailty and mortality. The selected 698 CpGs included loci in the genes involving immune activation and inflammatory processes, which is highly associated with HIV frailty and mortality. For example, the most significant smoking-associated gene, *AHRR*, not only involves the metabolism of endogenous toxins from smoking that result in pathological processes but also represses other signaling pathways, including *NF-κappaB*, and is capable of regulating inflammatory responses [38]. *TNFRSF4* has been shown to activate *NF-kappa B* and plays a role in apoptosis. In addition, a number of CpGs in the 698 CpG sites were previously reported to involve acceleration of aging, frailty, cancer pathogenesis, and all-cause mortality. Although the majority of DNA methylation differences at a single CpG site between smokers and non-smokers are modest, the 698 features were enriched in pathways highly relevant to disease prognosis, frailty, and mortality.

While a model of 698 CpG sites may seem to be a large number of features for the prediction of frailty, emerging evidence has demonstrated that DNA methylation at individual CpG sites on a complex trait is small (less than 10%) [39]. In our EWAS analysis, the effect size of single CpG sites on smoking was in a range of 1 to 13%. To predict a complex outcome such as frailty with a small number of CpG sites is highly unlikely. A recently published paper showed a panel of 200 to 1100 CpG sites predicting multiple complex traits including alcohol, smoking, HDL cholesterol, education, and death [40]. Thus, a panel of hundreds of CpG sites predicting complex traits is expected. However, methods to select more informative features and to potentially reduce the number of features in future studies are warranted.

We acknowledge several limitations of this study. A recent study suggests that mRNA and miRNA profiles showed the best prediction for cancer prognosis [23]. Integrating DNA methylation with other omic and clinical data may improve the predictive value and clinical utility of the predicting model. Due to methodological limitations, we were unable to build a model to predict the VACS index as a continuous variable, which may have better clinical utility. The study was conducted in a retrospective cohort and smoking was defined from self-report, which may introduce bias. Applying our predictive model using the 698 selected features in a prospective cohort is warranted to confirm the results. The mechanisms that underlie the selected CpG features on HIV progression remain to be defined. Future studies of smoking’s effects on DNA methylation in HIV-infected specific cell types are warranted to better understand how the selected features involve smoking-related HIV prognosis.

Our results demonstrate a machine learning approach to establish methylation signatures for disease outcomes. The identified methylation sites may be a biological surrogate for the VACS index to measure clinical outcomes and to predict mortality. This first-ever methylation-based machine learning-based study sheds light on the impact of smoking on risk for complicated clinical outcomes, estimated using a molecular profile, in the setting of HIV infection.

## Conclusion

Applying DNA methylation-based ensemble learning, we identified a set of 698 smoking-associated DNA methylation CpG sites that predict HIV frailty and mortality. Building on more than 30 previous studies in HIV-uninfected persons, our findings suggest that smoking exposure changes DNA methylation in the HIV-infected host genome that is linked to HIV disease prognosis. Our results demonstrate that DNA methylation-based machine learning is a robust approach for the prediction of HIV prognosis.

## Methods

### Study population and phenotype assessment

The VACS, a nation-wide multicenter collaborative project designed to understand the role of co-morbid medical and psychiatric diseases in determining clinical outcomes in HIV infection, was the source of specimen and data (https://medicine.yale.edu/intmed/vacs/). The VACS biobank cohort is comprised of 2470 participants who were recruited for genetic studies from 2006 to 2007. Participants of the VACS biobank cohort provided written informed consent for the genetic study and provided blood samples. Clinical and demographic data were collected within 90 days of the blood sample collection. A total of 1137 samples were selected and randomly divided into two subsets (labeled cohort 1 and cohort 2), and DNA methylation was processed separately using different methylation arrays.

Self-report was used to collect information on smoking status. Current smokers were defined as smoking cigarettes daily during the past week; non-smokers reported never smoking cigarettes. The VACS created an index score to estimate overall frailty of HIV-infected individuals by summing pre-assigned points for age, routinely monitored indicators of HIV disease (CD4 count and HIV-1 RNA), and general indicators of organ system injury including hemoglobin, platelets, aspartate and alanine transaminase (AST and ALT), creatinine, and viral hepatitis C infection (HCV) (https://medicine.yale.edu/intmed/vacs/welcome/vacsindexinfo.aspx). The VACS index has been associated with important changes in health condition and behavior [41]. The VACS index has been shown to predict all-cause mortality among those undergoing treatment for HIV infection [42]. A higher VACS index score indicated greater frailty. Mortality rate 5 years after blood draw was 16%.

### Profiling DNA methylation using Illumina DNA methylation Beadchips

Genomic DNA was extracted from whole blood samples. DNA methylation profiling was conducted at the Yale Center for Genomic Analysis using the Illumina (San Diego, CA, USA) Infinium HumanMethylation450 BeadChip (HM450K) for cohort 1 and Illumina Infinium MethylationEPIC (EPIC) for cohort 2. Two sample sets were processed at different times but were processed by the same scientist at the Yale Center for Genomic Analysis who was blinded to the phenotypic information collected. All samples were randomly placed on each array and batch-corrected using the removeBatchEffect function in limma. Probe normalization and batch correction were performed as previously described by Lehne et al. [43].

#### Data quality control and normalization

In cohort 1, we removed 11,648 probes on sex chromosomes and 36,142 probes within 10 base pairs of single nucleotide polymorphisms. A total of 437,722 probes remained for analysis. As described by Lehne et al. [43], 24,416 probes on Y chromosomes were applied to evaluate the detection *p* value. A *p* < 1E−12 was set as a detection *p* value threshold to improve the quantification of methylation intensities. The intensity values with detection *p* > 1E−12 were labeled missing, and samples with a call rate < 98% were excluded. We also compared the predicted sex with self-reported sex. All samples matched as male. In cohort 2, we applied the same criteria for quality control. We removed 11 samples due to mismatched sex or low call rate. Only the 408,583 probes that were identical with HM450 array were extracted for replication analysis. Quantile normalization of intensity values was performed following the recommendations of Lehne et al. Six cell types (CD4+ T cells, CD8+ T cells, NK T cells, B cells, monocytes, and granulocytes) in the blood were estimated in each sample using the method described by Houseman et al. [44].

### Data analysis

The study design and analytical approaches are summarized in Fig. 1.

#### Epigenome-wide association analysis

Analyses of discovery and replication stages were performed using the same pipeline [43]. To adjust for significant global confounding factors, we conducted two serial regression analyses to determine the associations between methylome-wide CpGs and smoking. The following steps were performed to correct for global co-variations that may confound specific DNA methylation in smoking.

1.  The first principal component analysis (PCA) was performed to evaluate the intensity values of positive control probes designed in HM450. Then, the first GLM was performed as follows:

$$ Mehtylation\beta \sim \mathrm{age}+\mathrm{race}+\mathrm{alcohol}+\mathrm{ARTadherence}+{\log}_{10}\mathrm{VL}+\mathrm{WBC}+\mathrm{CD}8\mathrm{Tcell}+\mathrm{CD}4\mathrm{Tcell}+\mathrm{granulocyte}+\mathrm{NK}+\mathrm{Bcell}+\mathrm{monocyte}+{\mathrm{PC}}_{\mathrm{ControlProbe}}1-30. $$

The residuals for each probe and the top 30 PCs of the first PCA were used to adjust for technical biases, particularly batch effects.

2) The second PCA was performed on the resulting regression residuals from the first model. The top 5 PCs of the second PCA were used to control for global biological confounders that cannot be directly captured in the model.
3) Final GLM model

$$ \mathrm{Methylation}\beta \sim \mathrm{smoking}+\mathrm{age}+\mathrm{race}+\mathrm{alcohol}+\mathrm{ARTadherence}+{\log}_{10}\mathrm{VL}+\mathrm{WBC}+\mathrm{CD}8\mathrm{Tcell}+\mathrm{CD}4\mathrm{Tcell}+\mathrm{Gran}+\mathrm{NK}+\mathrm{Bcell}+\mathrm{Mono}+{\mathrm{PC}}_{\mathrm{ControlProbe}}1-30+{\mathrm{PC}}_{\mathrm{Residual}}1-5 $$

The significance threshold was set at *p* < 1.0E−07, which is equivalent to the Bonferroni correction.

#### Meta-analysis

We conducted an EWAS meta-analysis by combining the data from the discovery (cohort 1) and replication (cohort 2) samples. Effect size and *p* values for each probe were obtained from analyses in cohort 1 and cohort 2 samples, respectively. We performed fixed-effects, inverse-variance meta-analysis, with scheme parameters of sample size and standard error by implementing the METAL (ver: 2010-02-08) program, combining summary statistics in two sample sets. We investigated heterogeneity in two sample sets using the *I*^2^ statistic.

### Machine learning prediction HIV prognosis

Considering the samples were processed at different times and platforms, batch effects were removed using the removeBatchEffect function in *limma* using R (ver. 3.32.10) before performing the machine learning prediction. To reduce redundant DNA methylation signals and noise for improving the prediction accuracy of HIV frailty, CpG sites with FDR < 0.5 from EWAS in cohort 1 were selected for machine learning. The samples in cohort 1 were randomly divided into a training set and a test set with a ratio of 8:2. We first built a model using the training set, in which each sample was labeled poor (VACS index > 50) or good prognosis (VACS index ≤ 50). We then tested the model by performing 10-fold cross-validation in the testing set, and the best-performed model was tested in an independent replication set.

#### Prediction model and validation

Machine learning GLMNET was used to build a prediction model. A total of 997 CpGs from EWAS (FDR < 0.1) were ranked based on an importance value for each CpG from GLMNET. The CpG sites were clustered as 21 groups from 2 to 997 sites using 50 CpG increments.

Tenfold cross-validation was performed in the training set to identify the best performing model. Additional machine learning methods were used to predict the best outcomes. GLMENT, SVM, RF, and XGBoost were performed separately. The parameters were fine-tuned by using R package caret (ver: 6.0-78) (https://libraries.io/cran/caret/6.0-78) for each algorithm. To avoid bias of each method, we used the ensemble method with R package caretEnsemble (ver: 2.0.0) (https://cran.r-project.org/web/packages/caretEnsemble/index.html) that constructed a new model by weighing the vote of each CpG from four machine learning methods.

The testing set was employed to evaluate the model by ROC analysis. The best pre-formed features were used to further validate the model in the independent testing set (cohort 2) using an ensemble-based method. Sensitivity, specificity, and AUC were used to assess model performance.

### Association of DNA methylation index with mortality

To examine whether the selected CpG site methylation was associated with mortality, we constructed a methylation index from the 698 CpG sites following the previous formula [45]. A separate index was constructed for hypomethylated and hypermethylated CpG sites, respectively.

$$ \mathrm{SI}=\frac{1}{n}\sum \limits_{j=1}^n Wcj\frac{\beta_j-\beta .{\mathrm{Mean}}_{\mathrm{non}-\mathrm{smoker}}}{\beta .{\mathrm{SD}}_{\mathrm{non}-\mathrm{smoker}}} $$

The association of the DNA methylation risk index with all-cause mortality was examined by Kaplan-Meier plots and log-rank tests in all samples. Cox regression model was then used to adjust for age, antiretroviral therapy adherence, HIV-1 load, and CD4 count. In the Cox regression model, the DNA methylation index score was a categorical variable (using the highest quartiles as the reference category) or a continuous variable (calculating HR for a decrease in DNA methylation by one standard deviation). Index_hypo_ and index_hyper_ were evaluated for the prediction of mortality separately.

### Gene enrichment analysis

Pathway and network analysis was conducted for the selected 698 CpG sites on 455 genes by employing the Ingenuity Pathway Analysis (IPA). For genes with multiple CpG sites, the lowest *p* value at the CpG site within a gene was used to represent the gene level significance. Significant pathways were defined at a FDR < 0.05.

## Additional files

Additional file 1: **Table S1.** Epigenome-wide significant CpG sites associated with tobacco smoking in a discovery sample. **Table S2.** Epigenome-wide significant CpG sites associated with tobacco smoking in a replication sample. **Table S3.** Machine learning selected 698 CpGs for the prediction of HIV frailty. (XLSX 77 kb)

Additional file 2: **Figure S1.** Meta-analysis of epigenome-wide association of smoking in HIV-infected samples. A. Manhattan plot of meta-analysis in two sample sets. Red line indicates Bonferroni-corrected epigenome-wide significance; B. Hypo- and hyper-CpG sites associated with tobacco smoking. (PDF 1562 kb)

Additional file 3: **Figure S2.** Prediction of 408,583 CpG sites on HIV frailty by using GLMNET model. HIV frailty is represented by Veteran Aging Cohort Study index (VACS index). AUC: area under curve from receiver operating characteristic analysis. (PDF 54 kb)

Additional file 4: **Figure S3.** Prediction of smoking status on HIV frailty indicated by Veteran Aging Cohort Study (VACS) index. AUC: area under curve from receiver operating characteristic analysis. (PDF 8 kb)

Additional file 5: **Figure S4.** A prediction of the smoking-associated 698 CpG sites for mortality in a HIV-positive population. AUC: area under curve. (PDF 708 kb)

## Abbreviations

AA: African-American
ART: Antiretroviral therapy
AUC: Area under curve
AUDIT: Alcohol Use Disorder Identification Test-C
Bagging: Bootstrap aggregating
CI: Confidence interval
EWAS: Epigenome-wide association study
GLMNET: Lasso and elastic-net regularized generalized linear models
HR: Hazard ratio
RF: Random forest
SVM: Support vector method
VACS: Veteran Aging Cohort Study
VL: Viral load
WBCs: White blood cells
