# Supplementary figures and images for: Machine learning selected smoking-associated DNA methylation signatures that predict HIV prognosis and mortality
Source: Clin Epigenetics. 2018 Dec 13;10:155. doi: 10.1186/s13148-018-0591-z (PMC6293604; doi:10.1186/s13148-018-0591-z)

**A**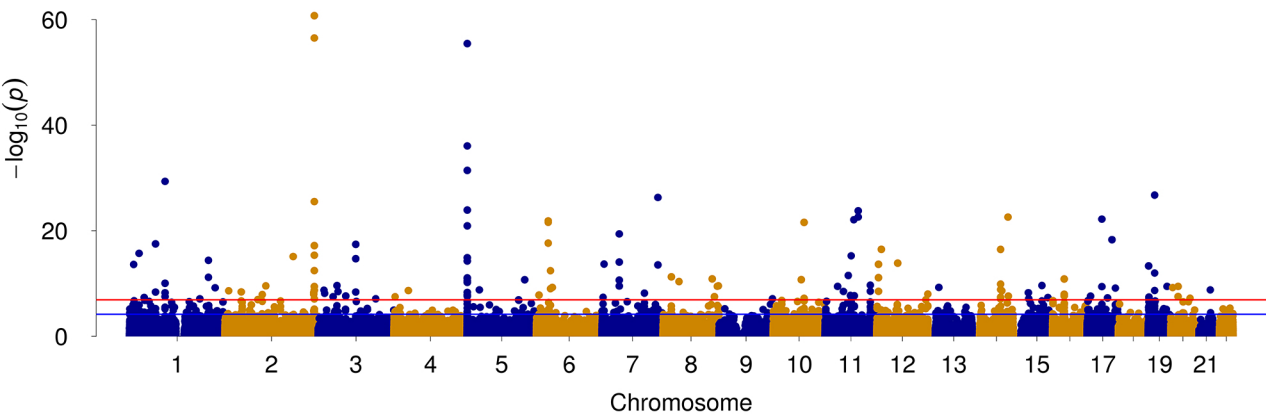**B**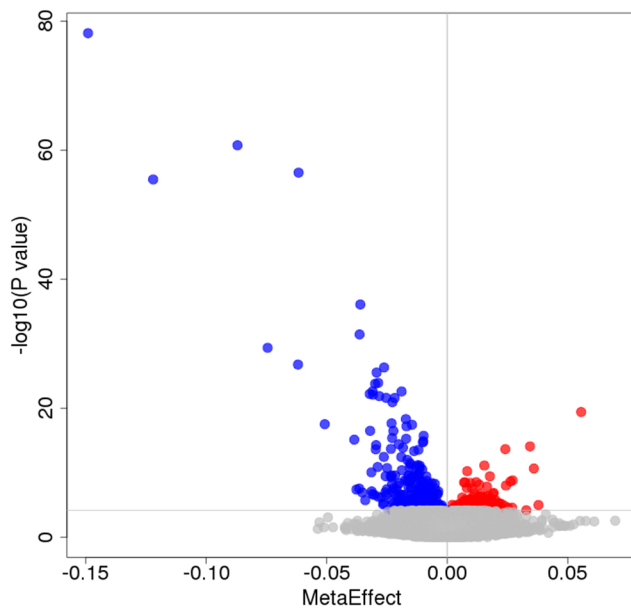

Supplement: Supplementary file 2 — Figure S1. Meta-analysis of epigenome-wide association of smoking in HIV-infected samples. A. Manhattan plot of meta-analysis in two sample sets. Red line indicates Bonferroni-corrected epigenome-wide significance; B. Hypo- and hyper-CpG sites associated with tobacco smoking. (PDF 1562 kb) [file 13148_2018_591_MOESM2_ESM.pdf]

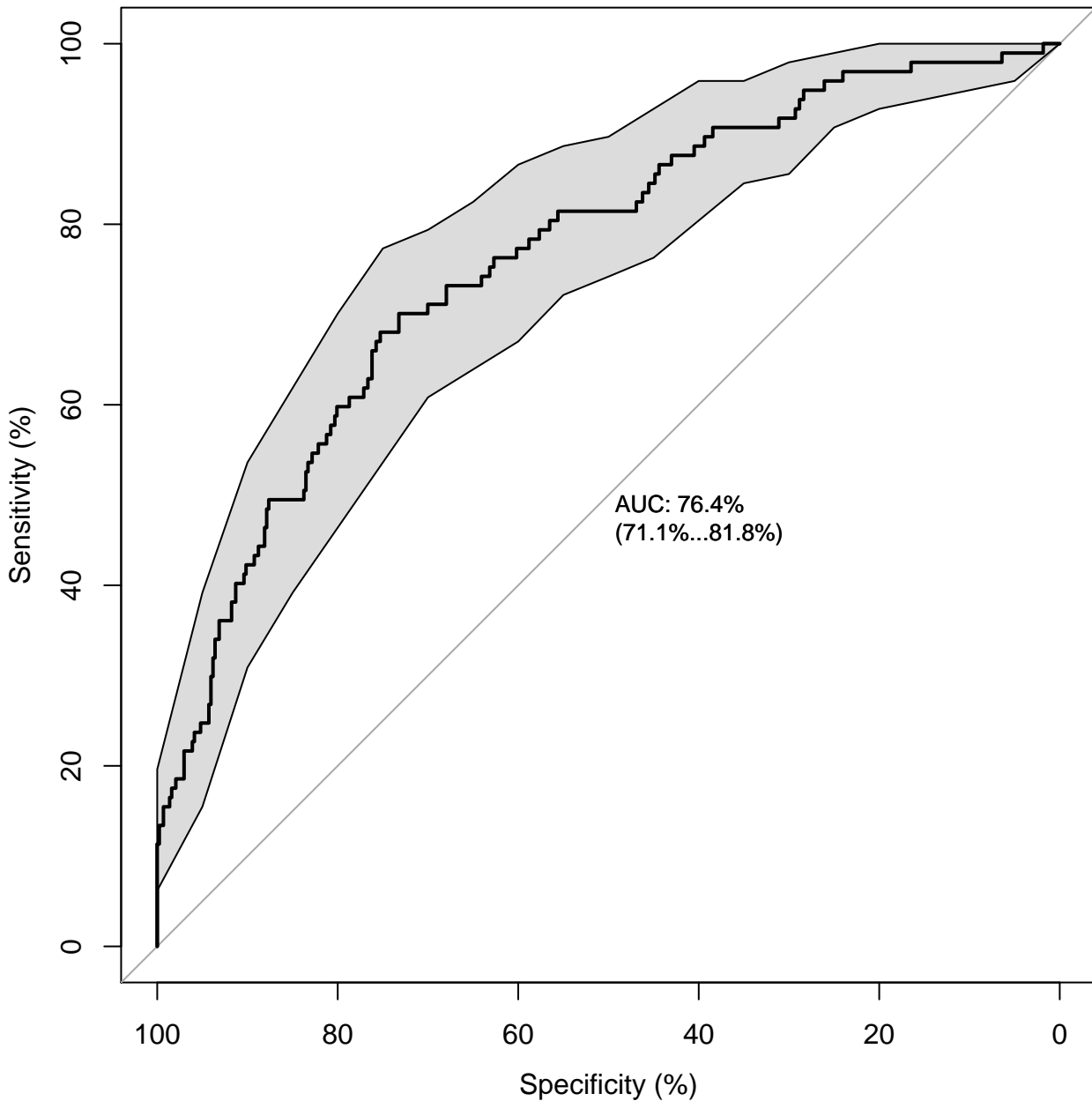

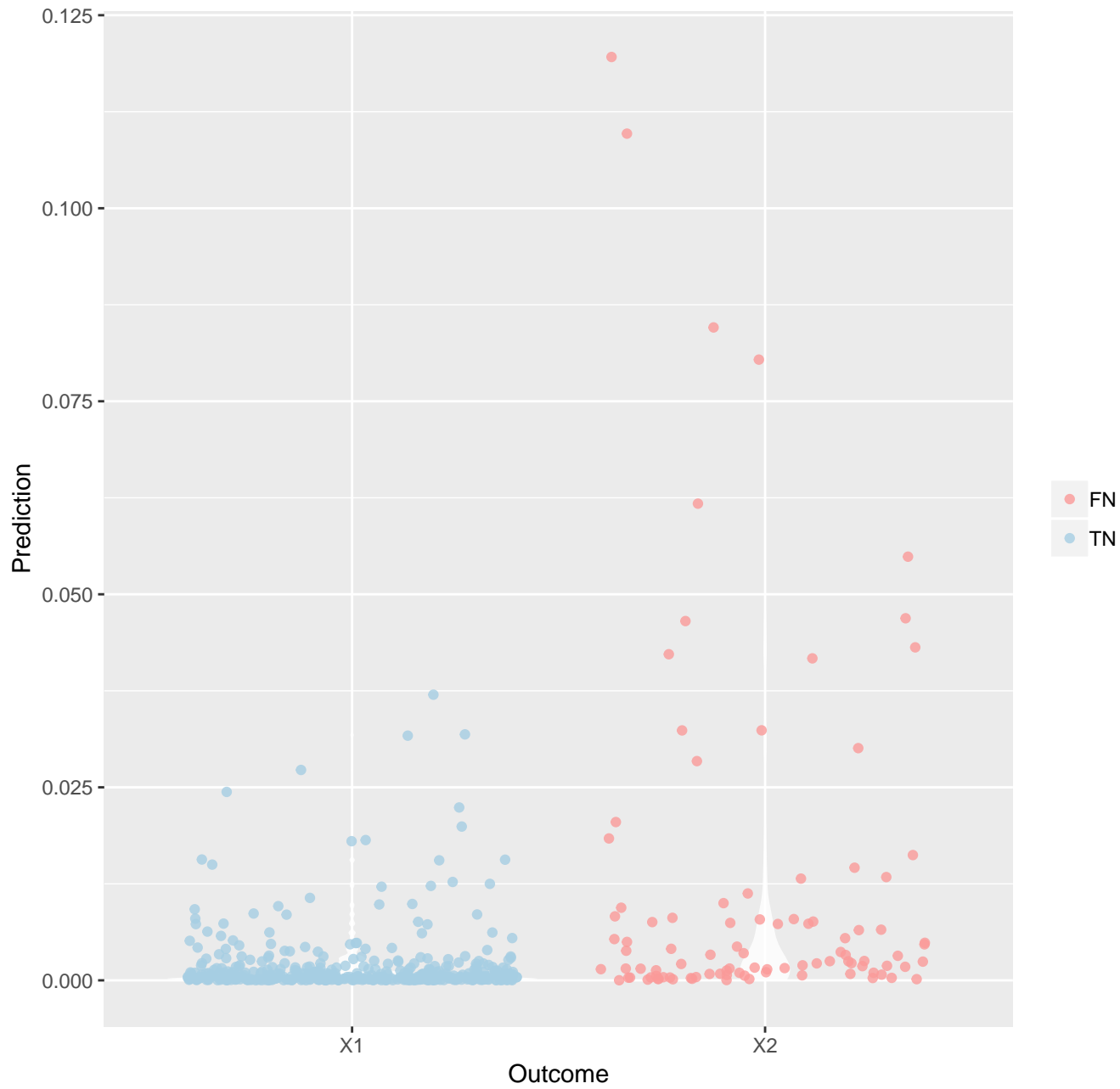

Supplement: Supplementary file 3 — Figure S2. Prediction of 408,583 CpG sites on HIV frailty by using GLMNET model. HIV frailty is represented by Veteran Aging Cohort Study index (VACS index). AUC: area under curve from receiver operating characteristic analysis. (PDF 54 kb) [file 13148_2018_591_MOESM3_ESM.pdf]

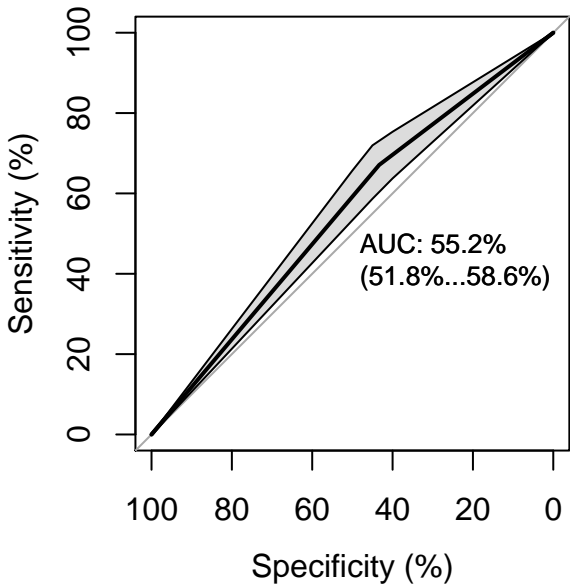

Supplement: Supplementary file 4 — Figure S3. Prediction of smoking status on HIV frailty indicated by Veteran Aging Cohort Study (VACS) index. AUC: area under curve from receiver operating characteristic analysis. (PDF 8 kb) [file 13148_2018_591_MOESM4_ESM.pdf]

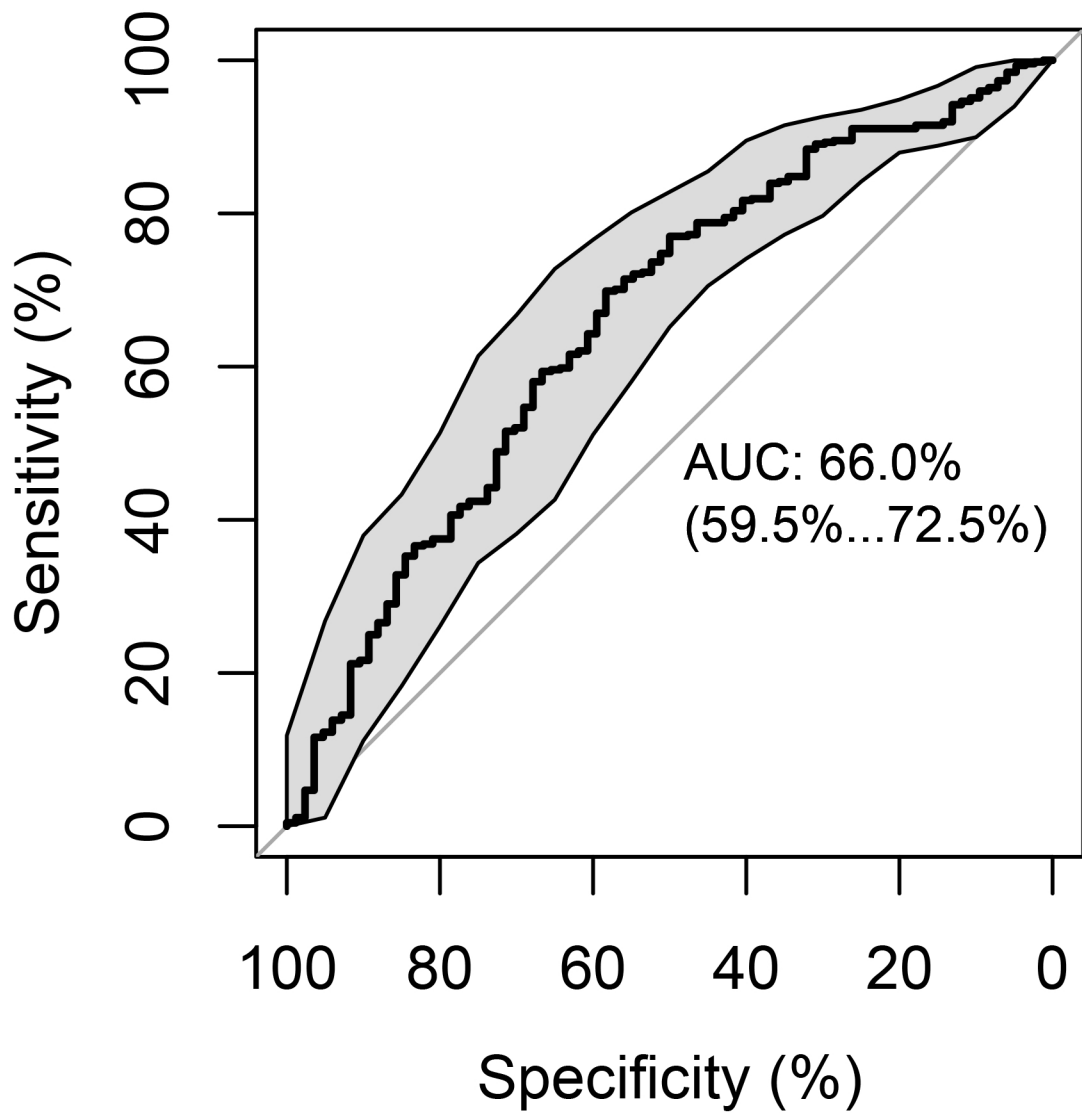

Supplement: Supplementary file 5 — Figure S4. A prediction of the smoking-associated 698 CpG sites for mortality in a HIV-positive population. AUC: area under curve. (PDF 708 kb) [file 13148_2018_591_MOESM5_ESM.pdf]
